# Eosinophilic Gastrointestinal Disease After Pediatric Organ and Bone Marrow Transplantation

**DOI:** 10.1002/jgh3.70344

**Published:** 2026-01-25

**Authors:** Erin Sinai, Bridget E. Wilson, Melissa Pecak, Shauna Schroeder, Edward L. Swing, Cindy Bauer

**Affiliations:** ^1^ School of Medicine, Creighton University Phoenix Arizona USA; ^2^ Division of Allergy and Immunology Phoenix Children's Phoenix Arizona USA; ^3^ Division of Allergy, Asthma, and Clinical Immunology Mayo Clinic Scottsdale Arizona USA; ^4^ Department of Gastroenterology Phoenix Children's Phoenix Arizona USA; ^5^ Department of Child Health University of Arizona College of Medicine Phoenix Arizona USA; ^6^ Graduate Medical Education, Phoenix Children's Phoenix Arizona USA

**Keywords:** allergy, eosinophilia, immune dysregulation, immunosuppression

## Abstract

**Background:**

Previous studies have shown an increased prevalence of eosinophilic gastrointestinal disease (EGID) in pediatric liver and heart transplant recipients. However, EGID after other solid organ and bone marrow transplantations has not been extensively evaluated.

**Aims:**

The purpose of this study is to determine relationships between subsets of EGID with different solid organ and bone marrow transplants in pediatric patients.

**Methods:**

We performed a single‐center retrospective chart review of pediatric patients with transplant and EGID between 2007 and 2023. For comparison between transplant groups, ANOVA was used for statistical analysis of continuous variables, and Fisher's exact test was used for categorical variables.

**Results:**

There were 30 patients with EGID (eosinophilic esophagitis [EoE] = 21; eosinophilic gastritis [EoG] = 4; EoE + EoG = 3; EoE + eosinophilic colitis [EoC] = 1; eosinophilic duodenitis [EoD] = 1) and history of transplant (liver = 15; heart = 9; bone marrow = 3; multivisceral liver + small bowel + pancreas = 2; kidney = 1). When comparing the transplant groups, there was a significant difference in EoE + EoG incidence (*p* = 0.011), specifically, EoE + EoG was present in 2 (100%) multivisceral and in 1/15 (7%) liver transplant patients. A statistically significant difference in the presence of gastroesophageal reflux (GER) and oral allergy syndrome between groups was noted (*p* = 0.036, *p* = 0.033). There was no significant difference in the symptoms leading to EGID work‐up: incidence; morphologic features; achievement of histologic remission; medications; or family history of atopy between groups.

**Conclusion:**

This retrospective biopsy‐confirmed cohort demonstrates that EGID subtype varies by transplant type, with higher rates of EoE + EoG in multivisceral recipients. Findings are exploratory and hypothesis‐generating; larger multicenter studies including more non‐liver and non‐heart transplant patients are needed.

## 1 Introduction

Eosinophilic gastrointestinal disease (EGID) represents a group of allergic inflammatory disorders of the gastrointestinal tract characterized by eosinophilic inflammation [1]. As the incidence of EGID in pediatric populations rises, there has been an even greater increase in EGID amongst post‐organ transplant pediatric patients [2]. This relationship between pediatric patients who underwent an organ transplant and subsequently developed EGID is thought to be due to mechanisms involving immune dysregulation due to immunosuppressive therapy, rejection episodes, food allergies, and viral infections [3, 4]. Previous studies have focused primarily on liver and heart transplant recipients, while differences in transplant type, immunosuppression, types of EGID, and remission treatments have not yet been explored [5, 6].

Pediatric liver transplant recipients that later developed EGID were found to have higher rates of comorbid IgE‐mediated food allergy, allergic rhinitis, asthma, and eczema, which could have put these patients at higher risk for later developing EGID post transplantation [5]. Further studies found a relationship between specific immunosuppressive medications, tacrolimus, and EGID incidence after liver transplantation [3, 5, 7, 8]. Specifically, tacrolimus may contribute to EGID by selectively suppressing Th1 lymphocytes while promoting the Th2 signal pathway, leading to an immune imbalance that enhances IL‐4 and IL‐5 production, driving eosinophilic recruitment and activation [9]. Similar findings were confirmed in pediatric heart transplantation studies [4, 9].

In this study from a single pediatric medical center, we investigated the relationship between various types of EGID in patients with bone marrow transplants (BMTs) and other solid organ transplants, including liver, heart, kidney, and multivisceral (liver, small bowel, and pancreas). The types of EGID analyzed include eosinophilic esophagitis (EoE), eosinophilic gastritis (EoG), eosinophilic duodenitis (EoD), and eosinophilic colitis (EoC), and combinations of the subtypes were included, including EoE + EoG and EoE + EoC. To our knowledge, this is the first study to characterize EGID in pediatric bone marrow and multivisceral transplant patients, providing novel insights into this complication in these populations. The purpose of this study is to determine relationships between subsets of EGID with different solid organ transplants and BMTs in pediatric patients.

## 2 Methods

This was an IRB‐approved (IRB‐23‐019) retrospective study of pediatric patients with solid organ transplant or BMT who were diagnosed with eosinophilic gastrointestinal disorders (EGID) at a medical center between 2007 and 2023. Patients were identified using ICD‐10 codes for EGID and transplant status: K52.81 (eosinophilic gastritis), K52.82 (eosinophilic colitis), Z94.0, Z94.1, Z94.2, Z94.3, Z94.4, Z94.81, Z94.82, Z94.83, Z94.84, and Z94.9. EGID was defined by established eosinophil thresholds: EoE ≥ 15 eos/hpf; EoG ≥ 30; EoD ≥ 52; right colon ≥ 100; transverse/descending colon ≥ 84; rectosigmoid ≥ 64 eos/hpf. Only biopsies meeting these thresholds were included. All data were de‐identified in REDCap. Pathology reviewers were blinded to transplant status. Patients were excluded if > 18 years, had a prior EGID diagnosis before transplant, or did not meet biopsy criteria. Diagnostic definitions were based on chart documentation and ICD‐10 coding. GER was identified through provider documentation and/or relevant ICD‐10 codes. ICD‐10 codes. Oral allergy syndrome (OAS) was defined by documented symptoms triggered by fresh fruits or vegetables, often accompanied by a food‐allergy ICD‐10 code. Histologic remission was defined as eosinophil counts below the site‐specific thresholds listed above. Medical records were reviewed to extract demographic and clinical data, including transplant type, EGID characteristics, and treatment history. Peripheral eosinophils were taken from available CBC/diff results. Because this was a retrospective review, timing was not standardized; we recorded the peak absolute eosinophil count documented for each patient. The age‐specific reference range at our institution is 0.05–0.70 × 10^9^/L. All patients were evaluated using a standardized clinical intake process, which included specific documentation of gastrointestinal symptoms (e.g., reflux, dysphagia, and abdominal pain) and relevant history, such as family history of atopy. Descriptive statistics were used to summarize the data for all patients. For comparisons between transplant groups, ANOVA was performed for continuous variables, and Fisher's exact test was used for categorical variables. All statistical analyses were conducted using SPSS statistical software (version 17.0).

## 3 Results

Thirty pediatric transplant recipients with EGID, including EoE, EoG, EoD, and EoC, and combinations of the subtypes were included, including EoE + EoG and EoE + EoC. Transplants included liver (*n* = 15), heart (*n* = 9), bone marrow (*n* = 3), multivisceral (*n* = 2), and kidney (*n* = 1). Of these, 47% were male and 40% were Hispanic. The mean age at the time of transplant was 37 months, with most patients having no atopic conditions before transplant (27/30, 90%). In general, most had no family history of atopy (21/30, 70%), and only 3 (10%) had a family history of EGID (Table 1). The average number of months between transplant and diagnostic esophagogastroduodenoscopy ± colonoscopy was 66.5 months, with the most common symptom leading to gastroenterology evaluation being vomiting (12/30, 40%). The average peak peripheral eosinophil count was 1.09 × 10^9^/L (range: 0.3–4.1). Additional demographic and baseline data are in Table 1.

**TABLE 1:** Demographics and pretransplant data.

Age	Months
Mean age at transplant, range	37.4 (1–226)
Sex	Number of patients (%)
Male	14 (47%)
Race	
Native American	2 (7%)
Asian	1 (3%)
Black	2 (7%)
White	25 (83%)
Ethnicity	
Hispanic	12 (40%)
Non‐Hispanic	16 (53%)
Not reported	2 (7%)
Medical insurance	
Private	19 (63%)
Atopic conditions before first transplant	
Asthma	1 (3%)
Allergic rhinitis ± conjunctivitis	1 (3%)
Atopic dermatitis	3 (10%)
None	27 (90%)
Family history of atopy	9 (30%)
Family history of EGID	3 (10%)
Gastrointestinal symptoms before first transplant	
Dysphagia or feeding dysfunction	2 (7%)
Abdominal pain	4 (13%)
Vomiting	4 (13%)
Diarrhea/loose stool	2 (7%)
Constipation	3 (10%)
Gastroesophageal reflux	2 (7%)
Poor weight gain/weight loss	3 (10%)
None	22 (73%)

**FIGURE 1:**
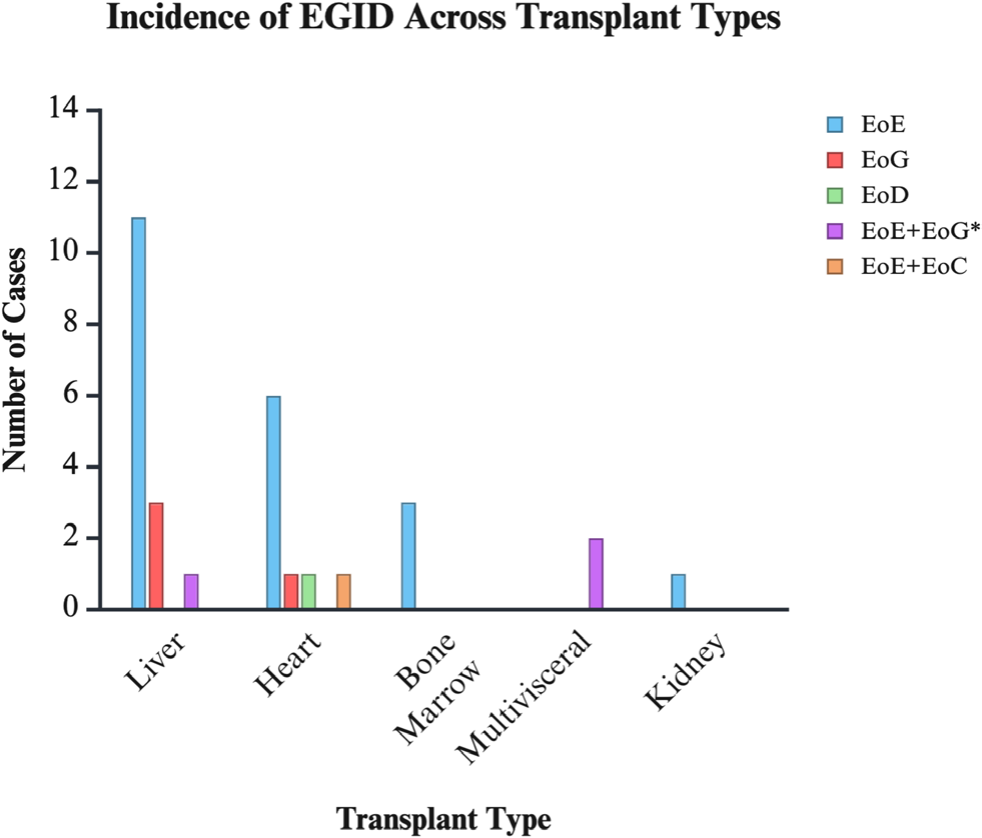
Incidence of EGID across different solid organ and bone marrow transplants in pediatric patients.

When comparing the various transplant groups, there was a significant difference in the incidence of EoE + EoG between all transplant groups, with EoE + EoG present in the 2 multivisceral transplant patients (*p* = 0.011; Table 2). These patients did not have underlying graft‐versus‐host disease (GVHD) or drug infections. One patient had post‐transplant lymphoproliferative disorder (PTLD). There were no other significant differences between EGID subtypes and transplant types (Figure 1).

**TABLE 2:** Summary of transplant and EGID data.

Transplant	Liver	Heart	Bone marrow	Multivisceral	Kidney	*p*
Number of patients, *n*	15	9	3	2	1	—
EGID, *n* (%)						
EoE	11 (73%)	6 (67%)	3 (100%)	0	1 (100%)	0.210
EoG	3 (20%)	1 (11%)	0	0	0	1.000
EoD	0	1 (11%)	0	0	0	0.500
EoE + EoG	1 (7%)	0	0	2 (100%)	0	0.011*
EoE + EoC	0	1 (11%)	0	0	0	0.500
Eosinophils/hpf on diagnostic scope, mean (SD)						
Upper esophagus	16.1 (14.2)	36.0 (31.9)	45.3 (41.1)	0.5 (0.7)	36	0.156
Middle esophagus	21.6 (39.2)	22.1 (27.2)	6.7 (11.5)	0	20	0.869
Lower esophagus	41.2 (25.8)	23.7 (25.3)	39.0 (14.9)	18.5 (2.1)	32	0.525
Gastric body	42.0 (44.4)	19.5 (27.6)	—	20.0 (28.3)	—	0.732
Gastric antrum	49.3 (43.5)	63.5 (51.6)	—	50.0 (70.7)	—	0.947
Morphologic features on diagnostic scope, *n* (%)						
Linear furrows	4 (27%)	3 (33%)	1 (33%)	0	0	1.000
White papules	1 (7%)	0	0	0	0	1.000
Edema	0	1 (11%)	1 (33%)	0	0	0.193
Erythema	0	0	1 (33%)	0	0	0.200
Erosion/ulceration	3 (20%)	0	0	0	0	0.634
Corrugated mucosa	1 (7%)	0	0	0	0	1.000
None	6 (40%)	6 (67%)	1 (33%)	2 (100%)	1 (100%)	0.369
Months between transplant and diagnostic scope, mean (SD)	64.9 (60.4)	56.7 (52.9)	65.0 (91.9)	97.0 (1.4)	90	0.894
Comorbid GI disorders, *n* (%)						
GER	1 (7%)	2 (22%)	2 (67%)	0	1 (100%)	0.036*
Celiac disease	0	0	0	1 (50%)	0	0.100
Inflammatory bowel disease	0	1 (11%)	0	1	0	0.131
Dysmotility/gastroparesis	1 (7%)	0	0	0	0	1.000
Rumination	1 (7%)	0	0	0	0	1.000
Chronic constipation	1 (7%)	0	1 (33%)	0	0	0.448
Enteral feeding tube	4 (27%)	5 (56%)	0	0	0	0.690
Short bowel	2 (13%)	0	0	1 (50%)	0	0.278
Gastroschisis	1 (7%)	0	0	0	0	1.000
GI bleed	0	0	0	1 (50%)	0	0.100
Total parenteral nutrition‐induced cholestasis	1 (7%)	0	0	0	0	1.000
Small intestinal bacterial overgrowth	1 (7%)	0	0	0	0	1.000
Post‐transplant lymphoproliferative disease of GI tract	0	1 (11%)	0	0	0	0.500
Comorbid atopic conditions, *n* (%)						
Allergic rhinitis ± conjunctivitis	6 (40%)	3 (33%)	2 (67%)	1 (50%)	1 (100%)	0.783
Atopic dermatitis	7 (47%)	3 (33%)	2 (67%)	0	0	0.688
IgE‐mediated food allergy	2 (13%)	0	0	0	0	0.690
Oral allergy syndrome	0	0	0	0	1 (100%)	0.033*
Asthma	3 (20%)	2 (22%)	1 (33%)	0	1 (100%)	0.471
Symptoms leading to GI evaluation, *n* (%)						
Dysphagia	2 (13%)	5 (56%)	1 (33%)	0	1 (100%)	0.079
Food impaction	1 (7%)	1 (11%)	0	0	0	1.000
Abdominal pain	5 (33%)	4 (44%)	1 (33%)	1 (50%)	0	0.954
Nausea	1 (7%)	0	1 (33%)	0	0	0.448
Vomiting	6 (40%)	4 (44%)	2 (67%)	0	0	0.754
Diarrhea/loose stool	3 (20%)	4 (44%)	1 (33%)	0	0	0.646
Poor weight gain/weight loss	5 (33%)	5 (56%)	0	0	1 (100%)	0.246
Food avoidance	4 (27%)	2 (22%)	0	0	0	1.000
Cough/difficulty breathing after eating	1 (7%)	0	0	0	1 (100%)	0.090
Bloating	1 (7%)	0	0	0	0	1.000

* Statistical significance.

A statistically significant difference in the presence of comorbid gastroesophageal reflux (GER) between groups was noted (*p* = 0.036; Table 2). Specifically, GER was present in one kidney (100%), one liver (1/15, 7%), two hearts (2/9, 22%), two bone marrows (2/3, 67%), and zero multivisceral transplant patients. OAS was diagnosed in the one kidney transplant patient but not in the other transplant patients (*p* = 0.033; Table 2).

Various immunosuppressant medications were used for induction and post‐solid organ transplant for the patients, including tacrolimus, mycophenolate, sirolimus, and glucocorticoids. Treatment with thymoglobulin during induction (*p* = 0.02; kidney *n* = 1; heart *n* = 4) and glucocorticoids during maintenance (*p* = 0.039; kidney *n* = 1, liver *n* = 3, heart *n* = 1, multivisceral *n* = 1) was associated with EoC incidence. For the other EGID subtypes, there was no association with specific induction or maintenance immunosuppressant medications. Similarly, there were no differences between morphologic features or eosinophils/high‐power field on diagnostic endoscopy; maximum recorded peripheral eosinophil count; achievement of histologic remission; medications leading to EGID remission; or family history of atopy or EGID. Additional results are summarized in Table 2.

## 4 Discussion

This study provides new insights into the incidence and characteristics of EGID in pediatric transplant recipients, showing significant associations with a variety of transplant types and comorbid conditions. Specifically, we found significant differences in the incidence of EoE + EoG between groups, with increased prevalence in the multivisceral transplant population. We also observed that thymoglobulin use during induction and glucocorticoid use during maintenance were associated with EoC incidence. This uniquely highlights the importance of studying EGID in all types of pediatric transplant recipients in addition to liver and heart.

Previous studies describe the relationship between peripheral eosinophilia and EGID after pediatric transplants with immunosuppressant medications [8, 10]. Immunosuppressant medications, specifically tacrolimus, have been linked to increased eosinophil production through differential inhibition of cytokine elaboration, which might alter the balance between Th1 and Th2 T‐cell subsets [7]. In contrast, in our study we found no differences in peripheral eosinophilia counts and EGID types with different immunosuppressant medications, including tacrolimus. However, we found that thymoglobulin use during induction and glucocorticoid use during maintenance had significant associations with EoC, while the exact mechanism for this association remains unclear. Previous studies have demonstrated a significant association between thymoglobulin and the development of PTLD in pediatric patients who have undergone bone marrow or multivisceral transplantation. While PTLD is distinct from EoC, both conditions may share underlying mechanisms related to immune dysregulation induced by immunosuppressive therapy. Thymoglobulin's potent T‐cell depletion may contribute to an imbalance in immune signaling, potentially favoring Th2‐driven eosinophilic inflammation in EGID while also creating an environment conducive to uncontrolled lymphoproliferation in PTLD [11, 12].

Previous studies have shown other causes of inflammation that lead to eosinophilia in transplant patients. These include drug reactions, infections, PTLD, and GVHD [3, 13]. Only one of our patients had comorbid PTLD, and no cases were attributed to drug interactions, infections, or GVHD. All diagnoses of EGID in our cohort were confirmed through endoscopy and histologic examination. Therefore, we can conclude that these cases of eosinophilia in our post‐transplant patients were related to EGID.

Ozdogan et al. demonstrated higher rates of allergen sensitization and atopy in liver transplant recipients with EGID when compared to those without EGID [2]. In our study, most patients did not have comorbid atopic conditions. This may be attributed to the younger median age at transplantation in our study population, which could mean that some patients had not yet developed atopic conditions at the time of analysis. The relationship between age at transplantation and the incidence of EGID has been explored in prior studies, with younger age identified as a risk factor for developing posttransplant allergic conditions, including EGID [2, 10]. The proposed mechanism involves immune dysregulation and an imbalance in immune signaling associated with early immune system development. Younger patients have an immature immune system that may be more prone to Th2‐skewed immune responses, leading to increased production of cytokines like IL‐4 and IL‐5, which drive eosinophilic recruitment and activation. In addition, early‐life immune disruptions, such as exposure to immunosuppressants like tacrolimus, may selectively suppress Th1 responses while promoting Th2 dominance, further enhancing allergic and eosinophilic inflammation. This heightened reactivity increases the risk of EGID development post‐transplant [2, 3, 6].

Clinically, these findings may help guide earlier screening in pediatric patients with EGID symptoms after solid organ and BMT. The association with immunosuppressant exposure suggests that immune modulation may play a role in disease mechanisms, although this requires further investigation. While exploratory, our results may inform future studies aimed at understanding potential inflammatory or immune‐mediated pathways.

This study has several limitations. First, its single‐center retrospective design introduces risks of selection and information bias, particularly because case identification relied on ICD‐10 code extraction and available chart documentation. The small sample size, especially in certain transplant groups, and imbalanced cohort distribution limit generalizability. Variability in pathologic criteria and biopsy availability, along with missing data on PPI use, dietary exposures, and avoidance history, may affect interpretation of eosinophil severity. Follow‐up duration also varied across patients, limiting assessment of longitudinal outcomes. Endoscopic evaluation was not performed uniformly, so the true incidence of EGID in transplant recipients remains unknown, and some cases may have been missed due to vague symptoms or immunosuppression blunting disease presentation. We were also unable to calculate incidence by transplant type without the total number of transplant recipients. Finally, this study is exploratory and identifies associations rather than causal relationships; mechanistic links, including those involving immunosuppressive agents, require further study.

## 5 Conclusion

To our knowledge, this is the largest cohort of multivisceral and BMT patients with EGID reported to date. In this population, we found all the patients with multivisceral transplants developed EoE + EoG, though a clear limitation is the small size of this group (*n* = 2). While previous reports describe an increase in the prevalence of post‐transplant allergy and autoimmune disorders in patients with multivisceral transplants, the relationship with EoE + EoG could be due to the altered immune response following multivisceral transplantation. Future studies including larger numbers of non‐liver and non‐heart transplant patients with EGID are needed to better understand the disease progression and chronicity of EGID in patients with transplantation. By broadening the scope of future studies to include diverse transplant populations, we can enhance our understanding and management of EGID in this unique group of patients.

## Author Contributions

**Erin Sinai:** data collection, drafting manuscript. **Bridget E. Wilson:** planning, drafting the manuscript. **Melissa Pecak:** data collection. **Shauna Schroeder:** planning, drafting the manuscript. **Edward L. Swing:** interpreting data. **Cindy Bauer:** planning, drafting the manuscript. All authors have approved the final draft being submitted.

## Funding

The authors have nothing to report.

## Ethics Statement

This study was approved by the IRB‐23‐019.

## Consent

Informed consent was obtained from legal guardians.

## Conflicts of Interest

The authors declare no conflicts of interest.

## Data Availability

The data that support the findings of this study are available from the corresponding author upon reasonable request.
